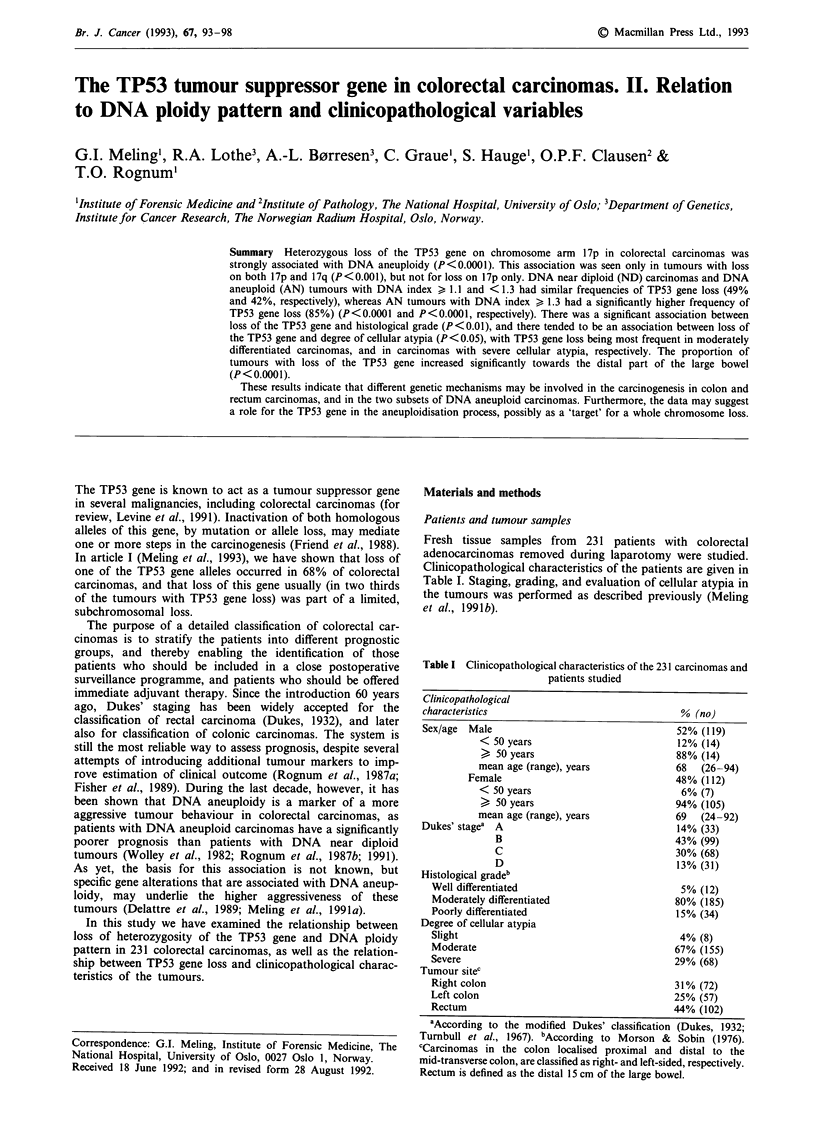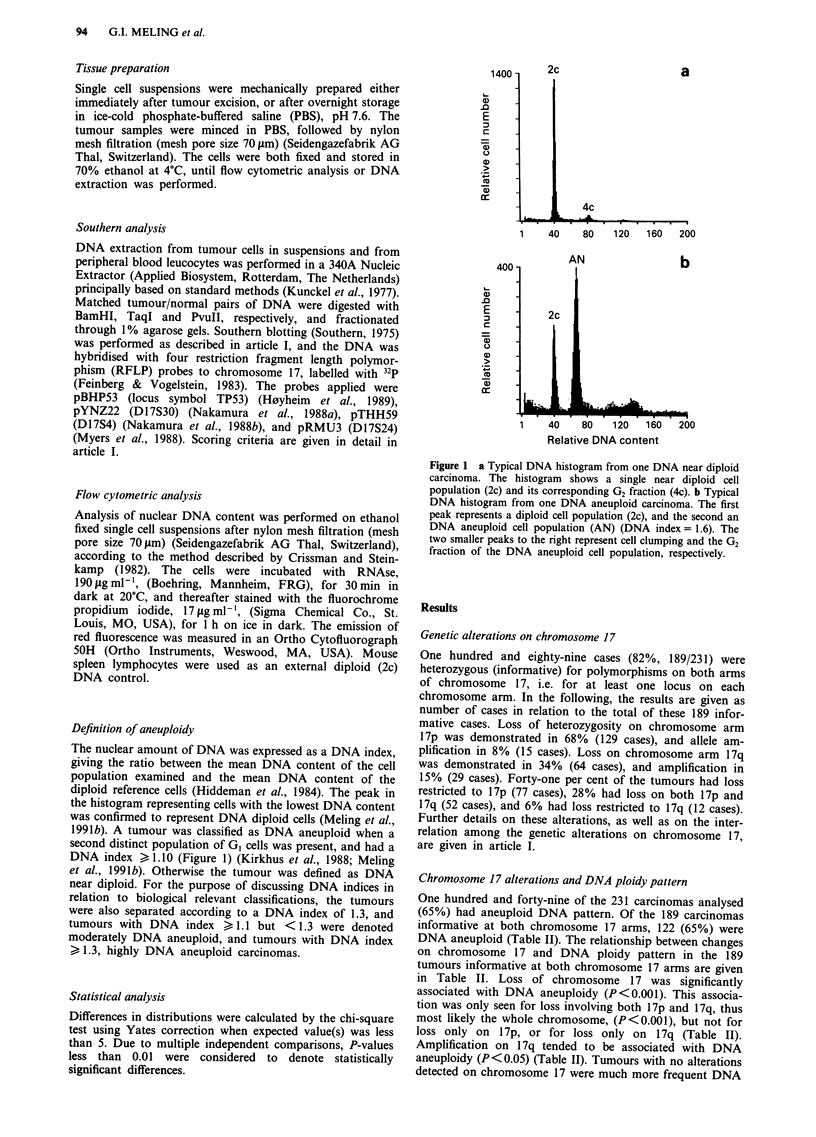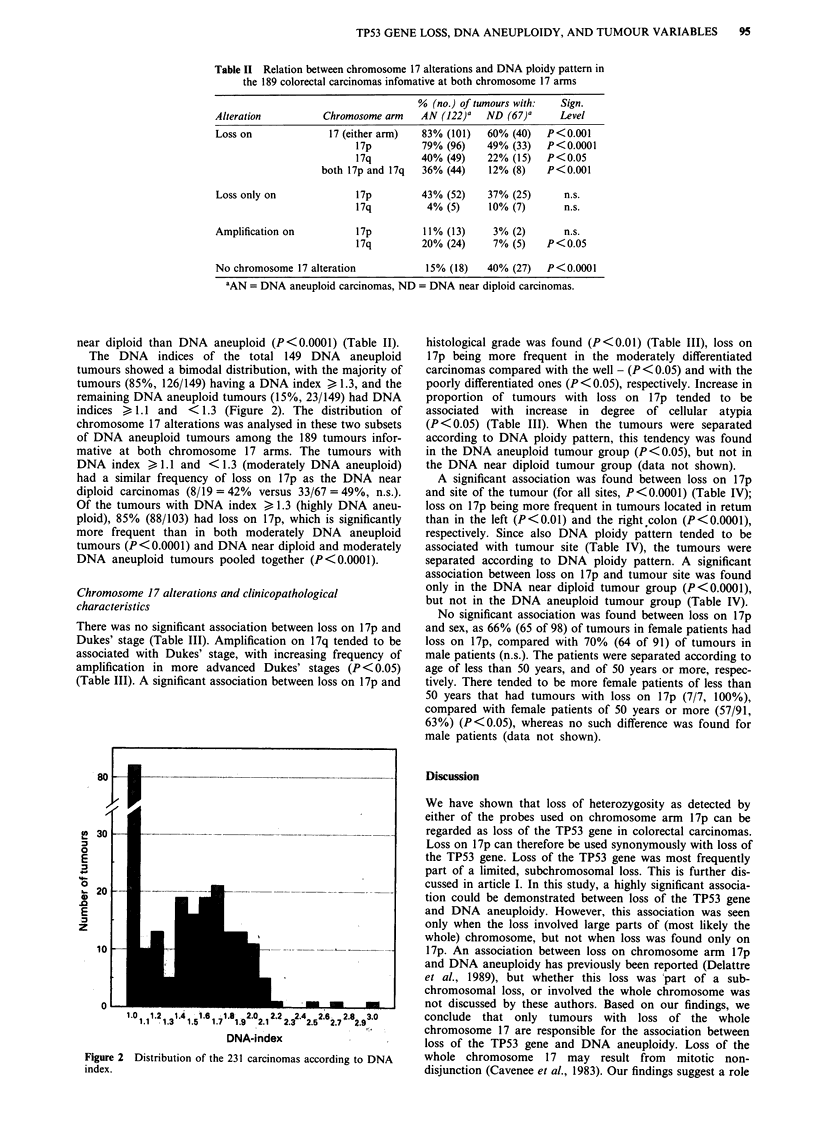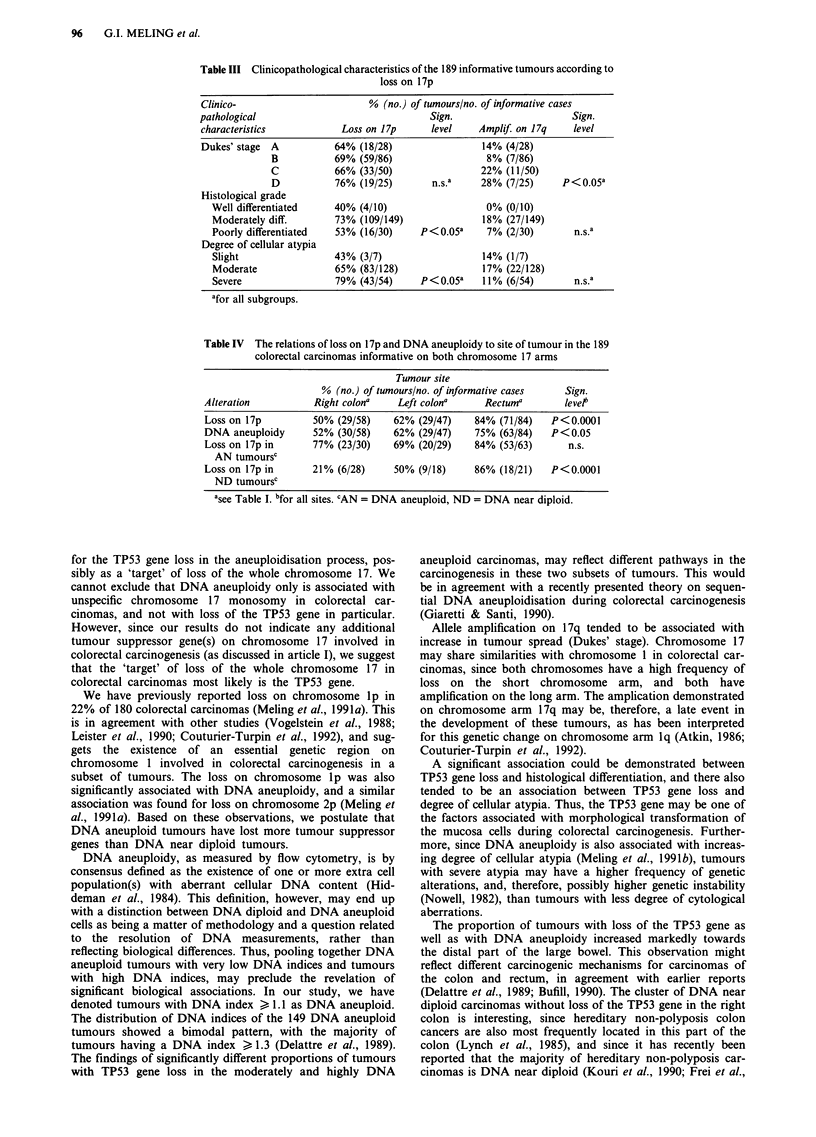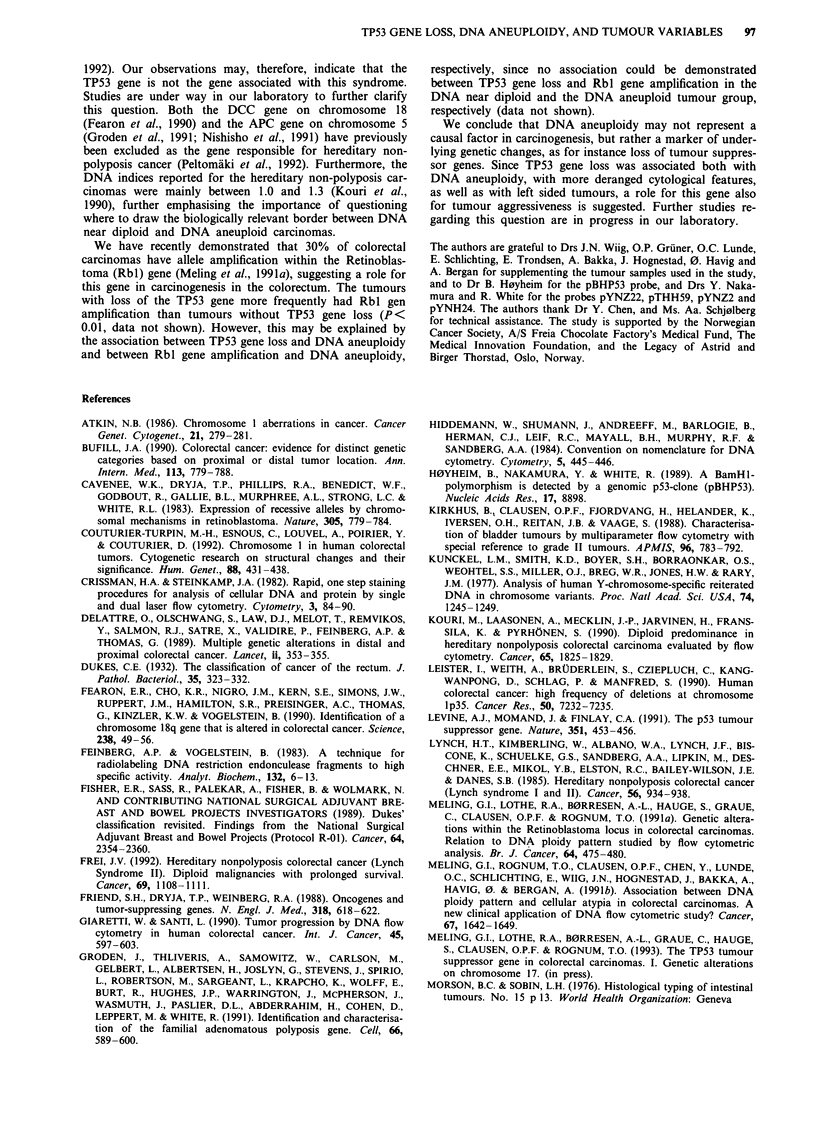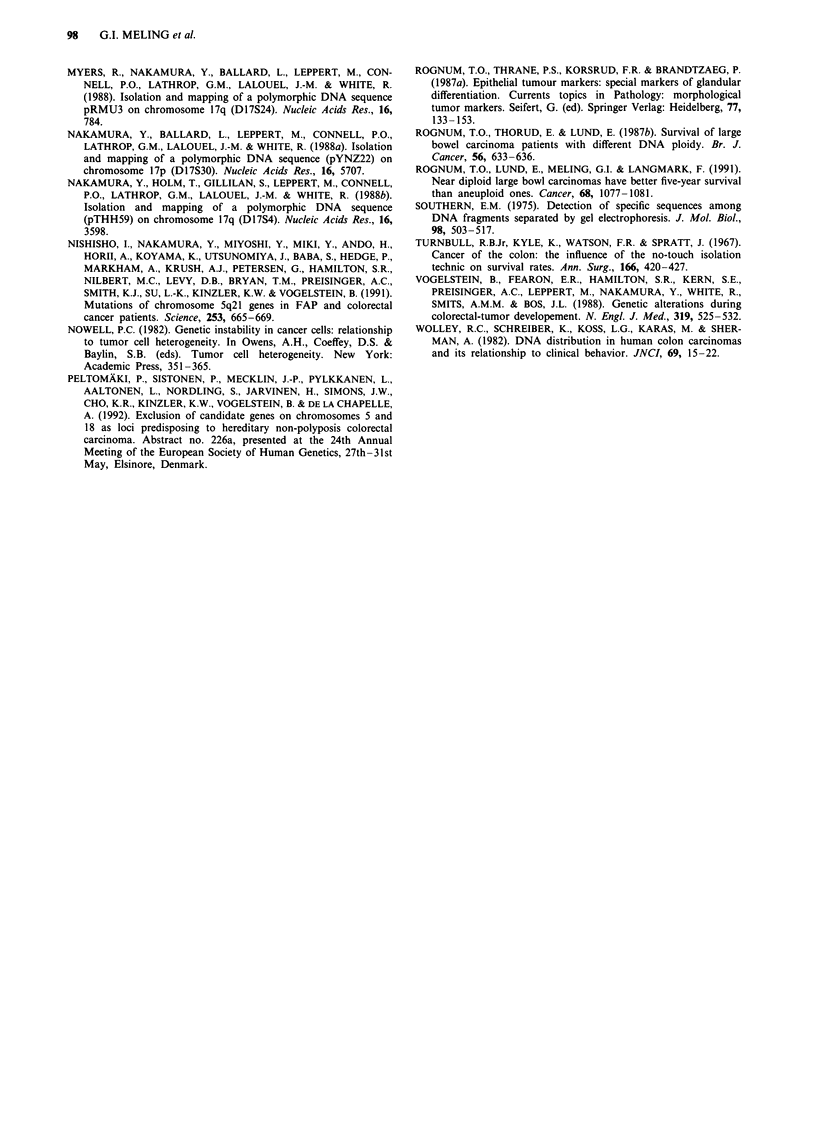# The TP53 tumour suppressor gene in colorectal carcinomas. II. Relation to DNA ploidy pattern and clinicopathological variables.

**DOI:** 10.1038/bjc.1993.15

**Published:** 1993-01

**Authors:** G. I. Meling, R. A. Lothe, A. L. Børresen, C. Graue, S. Hauge, O. P. Clausen, T. O. Rognum

**Affiliations:** Institute of Forensic Medicine, National Hospital, University of Oslo, Norway.

## Abstract

Heterozygous loss of the TP53 gene on chromosome arm 17p in colorectal carcinomas was strongly associated with DNA aneuploidy (P < 0.0001). This association was seen only in tumours with loss on both 17p and 17q (P < 0.001), but not for loss on 17p only. DNA near diploid (ND) carcinomas and DNA aneuploid (AN) tumours with DNA index > or = 1.1 and < 1.3 had similar frequencies of TP53 gene loss (49% and 42%, respectively), whereas AN tumours with DNA index > or = 1.3 had a significantly higher frequency of TP53 gene loss (85%) (P < 0.0001 and P < 0.0001, respectively). There was a significant association between loss of the TP53 gene and histological grade (P < 0.01), and there tended to be an association between loss of the TP53 gene and degree of cellular atypia (P < 0.05), with TP53 gene loss being most frequent in moderately differentiated carcinomas, and in carcinomas with severe cellular atypia, respectively. The proportion of tumours with loss of the TP53 gene increased significantly towards the distal part of the large bowel (P < 0.0001). These results indicate that different genetic mechanisms may be involved in the carcinogenesis in colon and rectum carcinomas, and in the two subsets of DNA aneuploid carcinomas. Furthermore, the data may suggest a role for the TP53 gene in the aneuploidisation process, possibly as a 'target' for a whole chromosome loss.


Br. J. Cancer (1993), 67, 93-98
© Macmillan Press Ltd., 1993

### The TP53 tumour suppressor gene in colorectal carcinomas. II. Relation to DNA ploidy pattern and clinicopathological variables

G.I. Meling[1], R.A. Lothe[3], A.-L. Børresen[3], C. Graue[1], S. Hauge[1], O.P.F. Clausen[2] & T.O. Rognum[1]

[1]*Institute of Forensic Medicine and* [2]*Institute of Pathology, The National Hospital, University of Oslo;* [3]*Department of Genetics, Institute for Cancer Research, The Norwegian Radium Hospital, Oslo, Norway.*

**Summary** Heterozygous loss of the TP53 gene on chromosome arm 17p in colorectal carcinomas was strongly associated with DNA aneuploidy ($P<0.0001$). This association was seen only in tumours with loss on both 17p and 17q ($P<0.001$), but not for loss on 17p only. DNA near diploid (ND) carcinomas and DNA aneuploid (AN) tumours with DNA index $\geqslant 1.1$ and $<1.3$ had similar frequencies of TP53 gene loss (49% and 42%, respectively), whereas AN tumours with DNA index $\geqslant 1.3$ had a significantly higher frequency of TP53 gene loss (85%) ($P<0.0001$ and $P<0.0001$, respectively). There was a significant association between loss of the TP53 gene and histological grade ($P<0.01$), and there tended to be an association between loss of the TP53 gene and degree of cellular atypia ($P<0.05$), with TP53 gene loss being most frequent in moderately differentiated carcinomas, and in carcinomas with severe cellular atypia, respectively. The proportion of tumours with loss of the TP53 gene increased significantly towards the distal part of the large bowel ($P<0.0001$).

These results indicate that different genetic mechanisms may be involved in the carcinogenesis in colon and rectum carcinomas, and in the two subsets of DNA aneuploid carcinomas. Furthermore, the data may suggest a role for the TP53 gene in the aneuploidisation process, possibly as a 'target' for a whole chromosome loss.

The TP53 gene is known to act as a tumour suppressor gene in several malignancies, including colorectal carcinomas (for review, Levine *et al.*, 1991). Inactivation of both homologous alleles of this gene, by mutation or allele loss, may mediate one or more steps in the carcinogenesis (Friend *et al.*, 1988). In article I (Meling *et al.*, 1993), we have shown that loss of one of the TP53 gene alleles occurred in 68% of colorectal carcinomas, and that loss of this gene usually (in two thirds of the tumours with TP53 gene loss) was part of a limited, subchromosomal loss.

The purpose of a detailed classification of colorectal carcinomas is to stratify the patients into different prognostic groups, and thereby enabling the identification of those patients who should be included in a close postoperative surveillance programme, and patients who should be offered immediate adjuvant therapy. Since the introduction 60 years ago, Dukes' staging has been widely accepted for the classification of rectal carcinoma (Dukes, 1932), and later also for classification of colonic carcinomas. The system is still the most reliable way to assess prognosis, despite several attempts of introducing additional tumour markers to improve estimation of clinical outcome (Rognum *et al.*, 1987*a*; Fisher *et al.*, 1989). During the last decade, however, it has been shown that DNA aneuploidy is a marker of a more aggressive tumour behaviour in colorectal carcinomas, as patients with DNA aneuploid carcinomas have a significantly poorer prognosis than patients with DNA near diploid tumours (Wolley *et al.*, 1982; Rognum *et al.*, 1987*b*; 1991). As yet, the basis for this association is not known, but specific gene alterations that are associated with DNA aneuploidy, may underlie the higher aggressiveness of these tumours (Delattre *et al.*, 1989; Meling *et al.*, 1991*a*).

In this study we have examined the relationship between loss of heterozygosity of the TP53 gene and DNA ploidy pattern in 231 colorectal carcinomas, as well as the relationship between TP53 gene loss and clinicopathological characteristics of the tumours.

Correspondence: G.I. Meling, Institute of Forensic Medicine, The National Hospital, University of Oslo, 0027 Oslo 1, Norway.
Received 18 June 1992; and in revised form 28 August 1992.

### Materials and methods

### *Patients and tumour samples*

Fresh tissue samples from 231 patients with colorectal adenocarcinomas removed during laparotomy were studied. Clinicopathological characteristics of the patients are given in Table I. Staging, grading, and evaluation of cellular atypia in the tumours was performed as described previously (Meling *et al.*, 1991*b*).

**Table I** Clinicopathological characteristics of the 231 carcinomas and patients studied

| *Clinicopathological characteristics* | *% (no)* |
|---|---|
| Sex/age Male | 52% (119) |
| < 50 years | 12% (14) |
| ⩾ 50 years | 88% (14) |
| mean age (range), years | 68 (26–94) |
| Female | 48% (112) |
| < 50 years | 6% (7) |
| ⩾ 50 years | 94% (105) |
| mean age (range), years | 69 (24–92) |
| Dukes' stage[a] A | 14% (33) |
| B | 43% (99) |
| C | 30% (68) |
| D | 13% (31) |
| Histological grade[b] | |
| Well differentiated | 5% (12) |
| Moderately differentiated | 80% (185) |
| Poorly differentiated | 15% (34) |
| Degree of cellular atypia | |
| Slight | 4% (8) |
| Moderate | 67% (155) |
| Severe | 29% (68) |
| Tumour site[c] | |
| Right colon | 31% (72) |
| Left colon | 25% (57) |
| Rectum | 44% (102) |

[a]According to the modified Dukes' classification (Dukes, 1932; Turnbull *et al.*, 1967). [b]According to Morson & Sobin (1976). [c]Carcinomas in the colon localised proximal and distal to the mid-transverse colon, are classified as right- and left-sided, respectively. Rectum is defined as the distal 15 cm of the large bowel.

*Tissue preparation*

Single cell suspensions were mechanically prepared either immediately after tumour excision, or after overnight storage in ice-cold phosphate-buffered saline (PBS), pH 7.6. The tumour samples were minced in PBS, followed by nylon mesh filtration (mesh pore size 70 μm) (Seidengazefabrik AG Thal, Switzerland). The cells were both fixed and stored in 70% ethanol at 4°C, until flow cytometric analysis or DNA extraction was performed.

*Southern analysis*

DNA extraction from tumour cells in suspensions and from peripheral blood leucocytes was performed in a 340A Nucleic Extractor (Applied Biosystem, Rotterdam, The Netherlands) principally based on standard methods (Kunckel *et al.*, 1977). Matched tumour/normal pairs of DNA were digested with BamHI, TaqI and PvuII, respectively, and fractionated through 1% agarose gels. Southern blotting (Southern, 1975) was performed as described in article I, and the DNA was hybridised with four restriction fragment length polymorphism (RFLP) probes to chromosome 17, labelled with $^{32}P$ (Feinberg & Vogelstein, 1983). The probes applied were pBHP53 (locus symbol TP53) (Høyheim *et al.*, 1989), pYNZ22 (D17S30) (Nakamura *et al.*, 1988*a*), pTHH59 (D17S4) (Nakamura *et al.*, 1988*b*), and pRMU3 (D17S24) (Myers *et al.*, 1988). Scoring criteria are given in detail in article I.

*Flow cytometric analysis*

Analysis of nuclear DNA content was performed on ethanol fixed single cell suspensions after nylon mesh filtration (mesh pore size 70 μm) (Seidengazefabrik AG Thal, Switzerland), according to the method described by Crissman and Steinkamp (1982). The cells were incubated with RNAse, 190 μg ml$^{-1}$, (Boehring, Mannheim, FRG), for 30 min in dark at 20°C, and thereafter stained with the fluorochrome propidium iodide, 17 μg ml$^{-1}$, (Sigma Chemical Co., St. Louis, MO, USA), for 1 h on ice in dark. The emission of red fluorescence was measured in an Ortho Cytofluorograph 50H (Ortho Instruments, Weswood, MA, USA). Mouse spleen lymphocytes were used as an external diploid (2c) DNA control.

*Definition of aneuploidy*

The nuclear amount of DNA was expressed as a DNA index, giving the ratio between the mean DNA content of the cell population examined and the mean DNA content of the diploid reference cells (Hiddeman *et al.*, 1984). The peak in the histogram representing cells with the lowest DNA content was confirmed to represent DNA diploid cells (Meling *et al.*, 1991*b*). A tumour was classified as DNA aneuploid when a second distinct population of $G_1$ cells was present, and had a DNA index $\geqslant 1.10$ (Figure 1) (Kirkhus *et al.*, 1988; Meling *et al.*, 1991*b*). Otherwise the tumour was defined as DNA near diploid. For the purpose of discussing DNA indices in relation to biological relevant classifications, the tumours were also separated according to a DNA index of 1.3, and tumours with DNA index $\geqslant 1.1$ but $< 1.3$ were denoted moderately DNA aneuploid, and tumours with DNA index $\geqslant 1.3$, highly DNA aneuploid carcinomas.

*Statistical analysis*

Differences in distributions were calculated by the chi-square test using Yates correction when expected value(s) was less than 5. Due to multiple independent comparisons, *P*-values less than 0.01 were considered to denote statistically significant differences.

**Figure 1** **a** Typical DNA histogram from one DNA near diploid carcinoma. The histogram shows a single near diploid cell population (2c) and its corresponding $G_2$ fraction (4c). **b** Typical DNA histogram from one DNA aneuploid carcinoma. The first peak represents a diploid cell population (2c), and the second an DNA aneuploid cell population (AN) (DNA index = 1.6). The two smaller peaks to the right represent cell clumping and the $G_2$ fraction of the DNA aneuploid cell population, respectively.

### Results

*Genetic alterations on chromosome 17*

One hundred and eighty-nine cases (82%, 189/231) were heterozygous (informative) for polymorphisms on both arms of chromosome 17, i.e. for at least one locus on each chromosome arm. In the following, the results are given as number of cases in relation to the total of these 189 informative cases. Loss of heterozygosity on chromosome arm 17p was demonstrated in 68% (129 cases), and allele amplification in 8% (15 cases). Loss on chromosome arm 17q was demonstrated in 34% (64 cases), and amplification in 15% (29 cases). Forty-one per cent of the tumours had loss restricted to 17p (77 cases), 28% had loss on both 17p and 17q (52 cases), and 6% had loss restricted to 17q (12 cases). Further details on these alterations, as well as on the interrelation among the genetic alterations on chromosome 17, are given in article I.

*Chromosome 17 alterations and DNA ploidy pattern*

One hundred and forty-nine of the 231 carcinomas analysed (65%) had aneuploid DNA pattern. Of the 189 carcinomas informative at both chromosome 17 arms, 122 (65%) were DNA aneuploid (Table II). The relationship between changes on chromosome 17 and DNA ploidy pattern in the 189 tumours informative at both chromosome 17 arms are given in Table II. Loss of chromosome 17 was significantly associated with DNA aneuploidy ($P < 0.001$). This association was only seen for loss involving both 17p and 17q, thus most likely the whole chromosome, ($P < 0.001$), but not for loss only on 17p, or for loss only on 17q (Table II). Amplification on 17q tended to be associated with DNA aneuploidy ($P < 0.05$) (Table II). Tumours with no alterations detected on chromosome 17 were much more frequent DNA

**Table II** Relation between chromosome 17 alterations and DNA ploidy pattern in the 189 colorectal carcinomas infomative at both chromosome 17 arms

| *Alteration* | *Chromosome arm* | *% (no.) of tumours with:* AN (122)[a] | ND (67)[a] | *Sign. Level* |
|---|---|---|---|---|
| Loss on | 17 (either arm) | 83% (101) | 60% (40) | $P<0.001$ |
| | 17p | 79% (96) | 49% (33) | $P<0.0001$ |
| | 17q | 40% (49) | 22% (15) | $P<0.05$ |
| | both 17p and 17q | 36% (44) | 12% (8) | $P<0.001$ |
| Loss only on | 17p | 43% (52) | 37% (25) | n.s. |
| | 17q | 4% (5) | 10% (7) | n.s. |
| Amplification on | 17p | 11% (13) | 3% (2) | n.s. |
| | 17q | 20% (24) | 7% (5) | $P<0.05$ |
| No chromosome 17 alteration | | 15% (18) | 40% (27) | $P<0.0001$ |

[a]AN = DNA aneuploid carcinomas, ND = DNA near diploid carcinomas.

near diploid than DNA aneuploid ($P<0.0001$) (Table II).

The DNA indices of the total 149 DNA aneuploid tumours showed a bimodal distribution, with the majority of tumours (85%, 126/149) having a DNA index ⩾1.3, and the remaining DNA aneuploid tumours (15%, 23/149) had DNA indices ⩾1.1 and <1.3 (Figure 2). The distribution of chromosome 17 alterations was analysed in these two subsets of DNA aneuploid tumours among the 189 tumours informative at both chromosome 17 arms. The tumours with DNA index ⩾1.1 and <1.3 (moderately DNA aneuploid) had a similar frequency of loss on 17p as the DNA near diploid carcinomas (8/19 = 42% versus 33/67 = 49%, n.s.). Of the tumours with DNA index ⩾1.3 (highly DNA aneuploid), 85% (88/103) had loss on 17p, which is significantly more frequent than in both moderately DNA aneuploid tumours ($P<0.0001$) and DNA near diploid and moderately DNA aneuploid tumours pooled together ($P<0.0001$).

### *Chromosome 17 alterations and clinicopathological characteristics*

There was no significant association between loss on 17p and Dukes' stage (Table III). Amplification on 17q tended to be associated with Dukes' stage, with increasing frequency of amplification in more advanced Dukes' stages ($P<0.05$) (Table III). A significant association between loss on 17p and histological grade was found ($P<0.01$) (Table III), loss on 17p being more frequent in the moderately differentiated carcinomas compared with the well – ($P<0.05$) and with the poorly differentiated ones ($P<0.05$), respectively. Increase in proportion of tumours with loss on 17p tended to be associated with increase in degree of cellular atypia ($P<0.05$) (Table III). When the tumours were separated according to DNA ploidy pattern, this tendency was found in the DNA aneuploid tumour group ($P<0.05$), but not in the DNA near diploid tumour group (data not shown).

A significant association was found between loss on 17p and site of the tumour (for all sites, $P<0.0001$) (Table IV); loss on 17p being more frequent in tumours located in retum than in the left ($P<0.01$) and the right colon ($P<0.0001$), respectively. Since also DNA ploidy pattern tended to be associated with tumour site (Table IV), the tumours were separated according to DNA ploidy pattern. A significant association between loss on 17p and tumour site was found only in the DNA near diploid tumour group ($P<0.0001$), but not in the DNA aneuploid tumour group (Table IV).

No significant association was found between loss on 17p and sex, as 66% (65 of 98) of tumours in female patients had loss on 17p, compared with 70% (64 of 91) of tumours in male patients (n.s.). The patients were separated according to age of less than 50 years, and of 50 years or more, respectively. There tended to be more female patients of less than 50 years that had tumours with loss on 17p (7/7, 100%), compared with female patients of 50 years or more (57/91, 63%) ($P<0.05$), whereas no such difference was found for male patients (data not shown).

**Figure 2** Distribution of the 231 carcinomas according to DNA index.

### Discussion

We have shown that loss of heterozygosity as detected by either of the probes used on chromosome arm 17p can be regarded as loss of the TP53 gene in colorectal carcinomas. Loss on 17p can therefore be used synonymously with loss of the TP53 gene. Loss of the TP53 gene was most frequently part of a limited, subchromosomal loss. This is further discussed in article I. In this study, a highly significant association could be demonstrated between loss of the TP53 gene and DNA aneuploidy. However, this association was seen only when the loss involved large parts of (most likely the whole) chromosome, but not when loss was found only on 17p. An association between loss on chromosome arm 17p and DNA aneuploidy has previously been reported (Delattre *et al.*, 1989), but whether this loss was part of a subchromosomal loss, or involved the whole chromosome was not discussed by these authors. Based on our findings, we conclude that only tumours with loss of the whole chromosome 17 are responsible for the association between loss of the TP53 gene and DNA aneuploidy. Loss of the whole chromosome 17 may result from mitotic non-disjunction (Cavenee *et al.*, 1983). Our findings suggest a role

**Table III** Clinicopathological characteristics of the 189 informative tumours according to loss on 17p

| *Clinico-pathological characteristics* | *% (no.) of tumours/no. of informative cases* Loss on 17p | *Sign. level* | *Amplif. on 17q* | *Sign. level* |
|---|---|---|---|---|
| Dukes' stage A | 64% (18/28) | | 14% (4/28) | |
| B | 69% (59/86) | | 8% (7/86) | |
| C | 66% (33/50) | | 22% (11/50) | |
| D | 76% (19/25) | n.s.[a] | 28% (7/25) | $P<0.05$[a] |
| Histological grade | | | | |
| Well differentiated | 40% (4/10) | | 0% (0/10) | |
| Moderately diff. | 73% (109/149) | | 18% (27/149) | |
| Poorly differentiated | 53% (16/30) | $P<0.05$[a] | 7% (2/30) | n.s.[a] |
| Degree of cellular atypia | | | | |
| Slight | 43% (3/7) | | 14% (1/7) | |
| Moderate | 65% (83/128) | | 17% (22/128) | |
| Severe | 79% (43/54) | $P<0.05$[a] | 11% (6/54) | n.s.[a] |

[a]for all subgroups.

**Table IV** The relations of loss on 17p and DNA aneuploidy to site of tumour in the 189 colorectal carcinomas informative on both chromosome 17 arms

| *Alteration* | *Tumour site % (no.) of tumours/no. of informative cases* Right colon[a] | *Left colon*[a] | *Rectum*[a] | *Sign. level*[b] |
|---|---|---|---|---|
| Loss on 17p | 50% (29/58) | 62% (29/47) | 84% (71/84) | $P<0.0001$ |
| DNA aneuploidy | 52% (30/58) | 62% (29/47) | 75% (63/84) | $P<0.05$ |
| Loss on 17p in AN tumours[c] | 77% (23/30) | 69% (20/29) | 84% (53/63) | n.s. |
| Loss on 17p in ND tumours[c] | 21% (6/28) | 50% (9/18) | 86% (18/21) | $P<0.0001$ |

[a]see Table I. [b]for all sites. [c]AN = DNA aneuploid, ND = DNA near diploid.

for the TP53 gene loss in the aneuploidisation process, possibly as a 'target' of loss of the whole chromosome 17. We cannot exclude that DNA aneuploidy only is associated with unspecific chromosome 17 monosomy in colorectal carcinomas, and not with loss of the TP53 gene in particular. However, since our results do not indicate any additional tumour suppressor gene(s) on chromosome 17 involved in colorectal carcinogenesis (as discussed in article I), we suggest that the 'target' of loss of the whole chromosome 17 in colorectal carcinomas most likely is the TP53 gene.

We have previously reported loss on chromosome 1p in 22% of 180 colorectal carcinomas (Meling *et al.*, 1991*a*). This is in agreement with other studies (Vogelstein *et al.*, 1988; Leister *et al.*, 1990; Couturier-Turpin *et al.*, 1992), and suggets the existence of an essential genetic region on chromosome 1 involved in colorectal carcinogenesis in a subset of tumours. The loss on chromosome 1p was also significantly associated with DNA aneuploidy, and a similar association was found for loss on chromosome 2p (Meling *et al.*, 1991*a*). Based on these observations, we postulate that DNA aneuploid tumours have lost more tumour suppressor genes than DNA near diploid tumours.

DNA aneuploidy, as measured by flow cytometry, is by consensus defined as the existence of one or more extra cell population(s) with aberrant cellular DNA content (Hiddeman *et al.*, 1984). This definition, however, may end up with a distinction between DNA diploid and DNA aneuploid cells as being a matter of methodology and a question related to the resolution of DNA measurements, rather than reflecting biological differences. Thus, pooling together DNA aneuploid tumours with very low DNA indices and tumours with high DNA indices, may preclude the revelation of significant biological associations. In our study, we have denoted tumours with DNA index $\geqslant 1.1$ as DNA aneuploid. The distribution of DNA indices of the 149 DNA aneuploid tumours showed a bimodal pattern, with the majority of tumours having a DNA index $\geqslant 1.3$ (Delattre *et al.*, 1989). The findings of significantly different proportions of tumours with TP53 gene loss in the moderately and highly DNA aneuploid carcinomas, may reflect different pathways in the carcinogenesis in these two subsets of tumours. This would be in agreement with a recently presented theory on sequential DNA aneuploidisation during colorectal carcinogenesis (Giaretti & Santi, 1990).

Allele amplification on 17q tended to be associated with increase in tumour spread (Dukes' stage). Chromosome 17 may share similarities with chromosome 1 in colorectal carcinomas, since both chromosomes have a high frequency of loss on the short chromosome arm, and both have amplification on the long arm. The amplication demonstrated on chromosome arm 17q may be, therefore, a late event in the development of these tumours, as has been interpreted for this genetic change on chromosome arm 1q (Atkin, 1986; Couturier-Turpin *et al.*, 1992).

A significant association could be demonstrated between TP53 gene loss and histological differentiation, and there also tended to be an association between TP53 gene loss and degree of cellular atypia. Thus, the TP53 gene may be one of the factors associated with morphological transformation of the mucosa cells during colorectal carcinogenesis. Furthermore, since DNA aneuploidy is also associated with increasing degree of cellular atypia (Meling *et al.*, 1991*b*), tumours with severe atypia may have a higher frequency of genetic alterations, and, therefore, possibly higher genetic instability (Nowell, 1982), than tumours with less degree of cytological aberrations.

The proportion of tumours with loss of the TP53 gene as well as with DNA aneuploidy increased markedly towards the distal part of the large bowel. This observation might reflect different carcinogenic mechanisms for carcinomas of the colon and rectum, in agreement with earlier reports (Delattre *et al.*, 1989; Bufill, 1990). The cluster of DNA near diploid carcinomas without loss of the TP53 gene in the right colon is interesting, since hereditary non-polyposis colon cancers are also most frequently located in this part of the colon (Lynch *et al.*, 1985), and since it has recently been reported that the majority of hereditary non-polyposis carcinomas is DNA near diploid (Kouri *et al.*, 1990; Frei *et al.*,

1992). Our observations may, therefore, indicate that the TP53 gene is not the gene associated with this syndrome. Studies are under way in our laboratory to further clarify this question. Both the DCC gene on chromosome 18 (Fearon *et al.*, 1990) and the APC gene on chromosome 5 (Groden *et al.*, 1991; Nishisho *et al.*, 1991) have previously been excluded as the gene responsible for hereditary non-polyposis cancer (Peltomäki *et al.*, 1992). Furthermore, the DNA indices reported for the hereditary non-polyposis carcinomas were mainly between 1.0 and 1.3 (Kouri *et al.*, 1990), further emphasising the importance of questioning where to draw the biologically relevant border between DNA near diploid and DNA aneuploid carcinomas.

We have recently demonstrated that 30% of colorectal carcinomas have allele amplification within the Retinoblastoma (Rb1) gene (Meling *et al.*, 1991*a*), suggesting a role for this gene in carcinogenesis in the colorectum. The tumours with loss of the TP53 gene more frequently had Rb1 gen amplification than tumours without TP53 gene loss ($P < 0.01$, data not shown). However, this may be explained by the association between TP53 gene loss and DNA aneuploidy and between Rb1 gene amplification and DNA aneuploidy, respectively, since no association could be demonstrated between TP53 gene loss and Rb1 gene amplification in the DNA near diploid and the DNA aneuploid tumour group, respectively (data not shown).

We conclude that DNA aneuploidy may not represent a causal factor in carcinogenesis, but rather a marker of underlying genetic changes, as for instance loss of tumour suppressor genes. Since TP53 gene loss was associated both with DNA aneuploidy, with more deranged cytological features, as well as with left sided tumours, a role for this gene also for tumour aggressiveness is suggested. Further studies regarding this question are in progress in our laboratory.

The authors are grateful to Drs J.N. Wiig, O.P. Grüner, O.C. Lunde, E. Schlichting, E. Trondsen, A. Bakka, J. Hognestad, Ø. Havig and A. Bergan for supplementing the tumour samples used in the study, and to Dr B. Høyheim for the pBHP53 probe, and Drs Y. Nakamura and R. White for the probes pYNZ22, pTHH59, pYNZ2 and pYNH24. The authors thank Dr Y. Chen, and Ms. Aa. Schjølberg for technical assistance. The study is supported by the Norwegian Cancer Society, A/S Freia Chocolate Factory's Medical Fund, The Medical Innovation Foundation, and the Legacy of Astrid and Birger Thorstad, Oslo, Norway.

### References

ATKIN, N.B. (1986). Chromosome 1 aberrations in cancer. *Cancer Genet. Cytogenet.*, **21**, 279–281.

BUFILL, J.A. (1990). Colorectal cancer: evidence for distinct genetic categories based on proximal or distal tumor location. *Ann. Intern. Med.*, **113**, 779–788.

CAVENEE, W.K., DRYJA, T.P., PHILLIPS, R.A., BENEDICT, W.F., GODBOUT, R., GALLIE, B.L., MURPHREE, A.L., STRONG, L.C. & WHITE, R.L. (1983). Expression of recessive alleles by chromosomal mechanisms in retinoblastoma. *Nature*, **305**, 779–784.

COUTURIER-TURPIN, M.-H., ESNOUS, C., LOUVEL, A., POIRIER, Y. & COUTURIER, D. (1992). Chromosome 1 in human colorectal tumors. Cytogenetic research on structural changes and their significance. *Hum. Genet.*, **88**, 431–438.

CRISSMAN, H.A. & STEINKAMP, J.A. (1982). Rapid, one step staining procedures for analysis of cellular DNA and protein by single and dual laser flow cytometry. *Cytometry*, **3**, 84–90.

DELATTRE, O., OLSCHWANG, S., LAW, D.J., MELOT, T., REMVIKOS, Y., SALMON, R.J., SATRE, X., VALIDIRE, P., FEINBERG, A.P. & THOMAS, G. (1989). Multiple genetic alterations in distal and proximal colorectal cancer. *Lancet*, **ii**, 353–355.

DUKES, C.E. (1932). The classification of cancer of the rectum. *J. Pathol. Bacteriol.*, **35**, 323–332.

FEARON, E.R., CHO, K.R., NIGRO, J.M., KERN, S.E., SIMONS, J.W., RUPPERT, J.M., HAMILTON, S.R., PREISINGER, A.C., THOMAS, G., KINZLER, K.W. & VOGELSTEIN, B. (1990). Identification of a chromosome 18q gene that is altered in colorectal cancer. *Science*, **238**, 49–56.

FEINBERG, A.P. & VOGELSTEIN, B. (1983). A technique for radiolabeling DNA restriction endonculease fragments to high specific activity. *Analyt. Biochem.*, **132**, 6–13.

FISHER, E.R., SASS, R., PALEKAR, A., FISHER, B. & WOLMARK, N. AND CONTRIBUTING NATIONAL SURGICAL ADJUVANT BREAST AND BOWEL PROJECTS INVESTIGATORS (1989). Dukes' classification revisited. Findings from the National Surgical Adjuvant Breast and Bowel Projects (Protocol R-01). *Cancer*, **64**, 2354–2360.

FREI, J.V. (1992). Hereditary nonpolyposis colorectal cancer (Lynch Syndrome II). Diploid malignancies with prolonged survival. *Cancer*, **69**, 1108–1111.

FRIEND, S.H., DRYJA, T.P., WEINBERG, R.A. (1988). Oncogenes and tumor-suppressing genes. *N. Engl. J. Med.*, **318**, 618–622.

GIARETTI, W. & SANTI, L. (1990). Tumor progression by DNA flow cytometry in human colorectal cancer. *Int. J. Cancer*, **45**, 597–603.

GRODEN, J., THLIVERIS, A., SAMOWITZ, W., CARLSON, M., GELBERT, L., ALBERTSEN, H., JOSLYN, G., STEVENS, J., SPIRIO, L., ROBERTSON, M., SARGEANT, L., KRAPCHO, K., WOLFF, E., BURT, R., HUGHES, J.P., WARRINGTON, J., MCPHERSON, J., WASMUTH, J., PASLIER, D.L., ABDERRAHIM, H., COHEN, D., LEPPERT, M. & WHITE, R. (1991). Identification and characterisation of the familial adenomatous polyposis gene. *Cell*, **66**, 589–600.

HIDDEMANN, W., SHUMANN, J., ANDREEFF, M., BARLOGIE, B., HERMAN, C.J., LEIF, R.C., MAYALL, B.H., MURPHY, R.F. & SANDBERG, A.A. (1984). Convention on nomenclature for DNA cytometry. *Cytometry*, **5**, 445–446.

HØYHEIM, B., NAKAMURA, Y. & WHITE, R. (1989). A BamH1-polymorphism is detected by a genomic p53-clone (pBHP53). *Nucleic Acids Res.*, **17**, 8898.

KIRKHUS, B., CLAUSEN, O.P.F., FJORDVANG, H., HELANDER, K., IVERSEN, O.H., REITAN, J.B. & VAAGE, S. (1988). Characterisation of bladder tumours by multiparameter flow cytometry with special reference to grade II tumours. *APMIS*, **96**, 783–792.

KUNCKEL, L.M., SMITH, K.D., BOYER, S.H., BORRAONKAR, O.S., WEOHTEL, S.S., MILLER, O.J., BREG, W.R., JONES, H.W. & RARY, J.M. (1977). Analysis of human Y-chromosome-specific reiterated DNA in chromosome variants. *Proc. Natl Acad. Sci. USA*, **74**, 1245–1249.

KOURI, M., LAASONEN, A., MECKLIN, J.-P., JARVINEN, H., FRANSSILA, K. & PYRHÖNEN, S. (1990). Diploid predominance in hereditary nonpolyposis colorectal carcinoma evaluated by flow cytometry. *Cancer*, **65**, 1825–1829.

LEISTER, I., WEITH, A., BRÜDERLEIN, S., CZIEPLUCH, C., KANGWANPONG, D., SCHLAG, P. & MANFRED, S. (1990). Human colorectal cancer: high frequency of deletions at chromosome 1p35. *Cancer Res.*, **50**, 7232–7235.

LEVINE, A.J., MOMAND, J. & FINLAY, C.A. (1991). The p53 tumour suppressor gene. *Nature*, **351**, 453–456.

LYNCH, H.T., KIMBERLING, W., ALBANO, W.A., LYNCH, J.F., BISCONE, K., SCHUELKE, G.S., SANDBERG, A.A., LIPKIN, M., DESCHNER, E.E., MIKOL, Y.B., ELSTON, R.C., BAILEY-WILSON, J.E. & DANES, S.B. (1985). Hereditary nonpolyposis colorectal cancer (Lynch syndrome I and II). *Cancer*, **56**, 934–938.

MELING, G.I., LOTHE, R.A., BØRRESEN, A.-L., HAUGE, S., GRAUE, C., CLAUSEN, O.P.F. & ROGNUM, T.O. (1991*a*). Genetic alterations within the Retinoblastoma locus in colorectal carcinomas. Relation to DNA ploidy pattern studied by flow cytometric analysis. *Br. J. Cancer*, **64**, 475–480.

MELING, G.I., ROGNUM, T.O., CLAUSEN, O.P.F., CHEN, Y., LUNDE, O.C., SCHLICHTING, E., WIIG, J.N., HOGNESTAD, J., BAKKA, A., HAVIG, Ø. & BERGAN, A. (1991*b*). Association between DNA ploidy pattern and cellular atypia in colorectal carcinomas. A new clinical application of DNA flow cytometric study? *Cancer*, **67**, 1642–1649.

MELING, G.I., LOTHE, R.A., BØRRESEN, A.-L., GRAUE, C., HAUGE, S., CLAUSEN, O.P.F. & ROGNUM, T.O. (1993). The TP53 tumour suppressor gene in colorectal carcinomas. I. Genetic alterations on chromosome 17. (in press).

MORSON, B.C. & SOBIN, L.H. (1976). Histological typing of intestinal tumours. No. 15 p 13. *World Health Organization*: Geneva

MYERS, R., NAKAMURA, Y., BALLARD, L., LEPPERT, M., CONNELL, P.O., LATHROP, G.M., LALOUEL, J.-M. & WHITE, R. (1988). Isolation and mapping of a polymorphic DNA sequence pRMU3 on chromosome 17q (D17S24). *Nucleic Acids Res.*, **16**, 784.

NAKAMURA, Y., BALLARD, L., LEPPERT, M., CONNELL, P.O., LATHROP, G.M., LALOUEL, J.-M. & WHITE, R. (1988*a*). Isolation and mapping of a polymorphic DNA sequence (pYNZ22) on chromosome 17p (D17S30). *Nucleic Acids Res.*, **16**, 5707.

NAKAMURA, Y., HOLM, T., GILLILAN, S., LEPPERT, M., CONNELL, P.O., LATHROP, G.M., LALOUEL, J.-M. & WHITE, R. (1988*b*). Isolation and mapping of a polymorphic DNA sequence (pTHH59) on chromosome 17q (D17S4). *Nucleic Acids Res.*, **16**, 3598.

NISHISHO, I., NAKAMURA, Y., MIYOSHI, Y., MIKI, Y., ANDO, H., HORII, A., KOYAMA, K., UTSUNOMIYA, J., BABA, S., HEDGE, P., MARKHAM, A., KRUSH, A.J., PETERSEN, G., HAMILTON, S.R., NILBERT, M.C., LEVY, D.B., BRYAN, T.M., PREISINGER, A.C., SMITH, K.J., SU, L.-K., KINZLER, K.W. & VOGELSTEIN, B. (1991). Mutations of chromosome 5q21 genes in FAP and colorectal cancer patients. *Science*, **253**, 665-669.

NOWELL, P.C. (1982). Genetic instability in cancer cells: relationship to tumor cell heterogeneity. In Owens, A.H., Coeffey, D.S. & Baylin, S.B. (eds). Tumor cell heterogeneity. New York: Academic Press, 351-365.

PELTOMÄKI, P., SISTONEN, P., MECKLIN, J.-P., PYLKKANEN, L., AALTONEN, L., NORDLING, S., JARVINEN, H., SIMONS, J.W., CHO, K.R., KINZLER, K.W., VOGELSTEIN, B. & DE LA CHAPELLE, A. (1992). Exclusion of candidate genes on chromosomes 5 and 18 as loci predisposing to hereditary non-polyposis colorectal carcinoma. Abstract no. 226a, presented at the 24th Annual Meeting of the European Society of Human Genetics, 27th-31st May, Elsinore, Denmark.

ROGNUM, T.O., THRANE, P.S., KORSRUD, F.R. & BRANDTZAEG, P. (1987*a*). Epithelial tumour markers: special markers of glandular differentiation. Currents topics in Pathology: morphological tumor markers. Seifert, G. (ed). Springer Verlag: Heidelberg, **77**, 133-153.

ROGNUM, T.O., THORUD, E. & LUND, E. (1987*b*). Survival of large bowel carcinoma patients with different DNA ploidy. *Br. J. Cancer*, **56**, 633-636.

ROGNUM, T.O., LUND, E., MELING, G.I. & LANGMARK, F. (1991). Near diploid large bowl carcinomas have better five-year survival than aneuploid ones. *Cancer*, **68**, 1077-1081.

SOUTHERN, E.M. (1975). Detection of specific sequences among DNA fragments separated by gel electrophoresis. *J. Mol. Biol.*, **98**, 503-517.

TURNBULL, R.B.Jr, KYLE, K., WATSON, F.R. & SPRATT, J. (1967). Cancer of the colon: the influence of the no-touch isolation technic on survival rates. *Ann. Surg.*, **166**, 420-427.

VOGELSTEIN, B., FEARON, E.R., HAMILTON, S.R., KERN, S.E., PREISINGER, A.C., LEPPERT, M., NAKAMURA, Y., WHITE, R., SMITS, A.M.M. & BOS, J.L. (1988). Genetic alterations during colorectal-tumor developement. *N. Engl. J. Med.*, **319**, 525-532.

WOLLEY, R.C., SCHREIBER, K., KOSS, L.G., KARAS, M. & SHERMAN, A. (1982). DNA distribution in human colon carcinomas and its relationship to clinical behavior. *JNCI*, **69**, 15-22.